# Development of Wedelia (*Sphagneticola trilobata*) and Sembung Rambat (*Mikania micrantha*) Extracts as Herbal Medicine for Chronic Obstructive Pulmonary Disease (COPD)

**DOI:** 10.3390/cimb48070720

**Published:** 2026-07-15

**Authors:** Dyah Iswantini, Min Rahminiwati, Trivadila Trivadila, Novriyandi Hanif, Siti Sadiah, Rut Novalia Rahmawati Sianipar, Riska Amelia Candra, Rani Melati Sukma, Susi Indariani, Raisa Zahra, Muthia Khansa

**Affiliations:** 1 Department of Chemistry, Faculty of Mathematics and Natural Sciences, IPB University, Bogor 16680, West Java, Indonesia; trivadila@apps.ipb.ac.id (T.T.); nhanif@apps.ipb.ac.id (N.H.); rutnovalia@gmail.com (R.N.R.S.); riskaameliacandra30@gmail.com (R.A.C.); rani_melatisukma@apps.ipb.ac.id (R.M.S.); 2 Tropical Biopharmaca Research Center, IPB University, Bogor 16680, West Java, Indonesia; minrahminiwati@gmail.com (M.R.); sitisa@apps.ipb.ac.id (S.S.); susiin@apps.ipb.ac.id (S.I.); 3 School of Veterinary Medicine and Biomedical Sciences, IPB University, Bogor 16680, West Java, Indonesia; 4 Faculty of Medicine, Padjadjaran University, Bandung 45363, West Java, Indonesia; raisazahra03@gmail.com; 5 Faculty of Medicine, Universitas Indonesia, Jakarta 10430, Central Jakarta, Indonesia; muthiakhansa01@gmail.com

**Keywords:** anti-inflammation, COPD, herbal, *Mikania micrantha*, *Sphagneticola trilobata*

## Abstract

Chronic Obstructive Pulmonary Disease (COPD) is a chronic respiratory condition characterized by progressive deterioration in lung function and persistent symptoms, particularly dyspnea, which can lead to death. Inflammation is important in COPD pathogenesis because it causes persistent inflammatory responses in the respiratory tract. Therefore, this study aimed to investigate H_2_O extracts derived from Wedelia (*Sphagneticola trilobata*) and Sembung Rambat (*Mikania micrantha*), respectively, as anti-inflammatory agents and to analyze the metabolite profiles contained in the extracts. The results showed that Sembung Rambat H_2_O extract demonstrated significant inhibition of IL-2 (100 ± 0.00%), while Wedelia extract inhibited 74.68 ± 7.13% release of pro-inflammatory cytokine IL-6. Liquid Chromatography–Tandem Mass Spectrometry (LC-MS/MS) analysis mainly identified the presence of amino acids, phenolic acids, organic acids, and terpenoids in both extracts. These results suggest the promising potential of Wedelia and Sembung Rambat extracts as herbal therapy for COPD.

## 1. Introduction

The coronavirus SARS-CoV-2 is the root cause of COVID-19. The 2019–2020 coronavirus pandemic was caused by this illness. The pathogenesis is possibly related to the body’s hyperinflammatory response, which is identified by the presence of cytokine storm. COVID-19 patients may develop fever, dry cough, and more severe symptoms, including trouble breathing and chest tightness. The virus predominantly affects the respiratory system, which is already weakened in patients with Chronic Obstructive Pulmonary Disorder (COPD) [1,2].

Airflow from the lungs is blocked by COPD, which is a long-lasting inflammatory condition of the respiratory system. The primary physiological function of the lungs, the main organs of the respiratory system, is to enable the exchange of gases. WHO documented that COPD accounted for the third-highest mortality burden worldwide in 2019 [3]. COPD-related lung disease is caused by airway abnormalities (chronic bronchitis or bronchiolitis) and/or alveoli (emphysema). The primary sign of chronic bronchitis is a persistent cough, which is indicative of airway inflammation. Additionally, it prevents airflow by causing goblet cells to produce excessive amounts of mucus. On the other hand, emphysema causes alveolar damage and produces symptoms such as dyspnea. Alveolar septa disintegration, an increase in airspace volume, and a reduction in elasticity brought on by redox imbalance, as well as excessive inflammatory response, are the hallmarks of the pathophysiology. An inflammatory immune response or long-term inflammation of the lungs’ airways is brought on by exposure to dangerous particles and chemicals, especially those found in cigarette smoke. COPD’s pathophysiology is caused by this inflammatory reaction [4]. An increase in cells, including neutrophils and lymphocytes, as well as macrophages, causes the release of pro-inflammatory cytokines, including IL-6, TNF-α, IL-8, and IL-1β, as well as IL-2, which are mostly found in the lung parenchyma, peripheral airways, and pulmonary blood vessels, and typically indicate chronic inflammation [5,6]. Cytokines, in particular, are polypeptides that function as intercellular mediators. In respiratory tract illnesses, the generation of these inflammation-promoting cytokines adds to lung tissue damage and affects respiratory tract inflammation. Patients with COPD can be managed and treated using a variety of strategies. Oral medicines, like inhaled inflammation-inhibiting agents (corticosteroids) and bronchodilators, are prominent therapeutic options. Nevertheless, sustained corticosteroid therapy may predispose patients to multiple adverse clinical effects, including diabetes mellitus, skeletal demineralization, lens opacification, and an elevated susceptibility to infections [7,8,9,10].

Herbal plants contribute significantly to culture and traditional medicine practices in many countries. Indonesia is a megabiodiverse country with over 30,000 plant taxa that are suitable to be utilized as raw materials for herbal medicines. Flavonoids, terpenoids, alkaloids, and saponins are among the bioefficacious phytochemicals identified in plants of medicinal value that exhibit diverse pharmacodynamic properties. These compounds exhibit beneficial effects against different pathological conditions. Indonesians have locally used herbal plants as traditional medicines [11,12,13].

Globally, there is currently a growing trend toward a return to a traditional lifestyle, which includes the utilization of natural or herbal therapeutic compounds, with a prevalence of 88% [14]. The WHO advises utilizing herbs to preserve the health of the population. Globally, especially in Indonesia, the herbal product market is currently quite popular and has a lot of promise. With a market size estimated at USD 165.66 billion in 2021, the herbal products sector is forecast to attain a value of USD 5 trillion by 2050 [15,16].

A comparative study on three methods of treatment and management for COPD patients in China showed that the combination of treatment methods and Traditional Chinese Medicine (TCM) was the most successful method [17]. Several medicinal plants have been employed in China for COPD treatment, including milkvetch root (*Astragalus monholicus*). This plant reportedly improved immune function in patients with COPD who experienced acute aggravation [18].

Five Indonesian medicinal herbs have been demonstrated to decrease inflammatory regulation related to the respiratory system, according to a comprehensive review based on earlier publications. The plants are Sambiloto (*Andrographis paniculata*), Babadotan (*Ageratum conyzoides*), Kersen (*Muntingia calabura*), and Legetan Warak (*Adenostemma lavenia*), as well as Sembung Rambat (*Mikania micrantha Kunth*) [19]. An *A. paniculata* 98% ethanol extract inhibited the cytokines that promote inflammation release, like IL-1β and IL-6, as well as TNF-α, in LPS-treated RAW 264.7 cells in vitro, according to Lukito et al. [20]. *A. lavenia* aqueous extract attenuated IL-6 levels according to another in vitro work by Astuti et al. [21]. Additionally, an M. calabura ethanol-based extract reduced the activity of cyclooxygen-ase-2 (COX-2) [22]. Specifically, COX-2 is an enzyme that produces prostaglandins, which cause inflammation [19].

In vivo models have demonstrated several plant-derived extracts’ inflammation-modulating activity. In a hamster model, an *A. paniculata* extract intervention markedly decreased IL-6 secretion and expression in pulmonary tissue, as reported by Kongsomros et al. [6]. Likewise, Widyaningrum et al. [23] observed that an *M. calabura* ethanolic extract exhibited significant inflammation-reducing activity by reducing carrageenan-induced edema in mice within 4 h after induction with 1% carrageenan. Furthermore, Xu et al. [24] demonstrated that *A. conyzoides* extract suppressed the activation of the NLRP3 inflammasome pathway in a murine model of peritonitis. Additionally, according to Deori et al. [25], an *M. micrantha* ethanolic extract exhibited inflammation-reducing activity potential by lowering rat paw edema in acute and sub-acute inflammation caused by carrageenan. The compounds 1a,9b-dihydro-1H-cyclopropa[a]anthracene, found in *A. lavenia* extract, and 7-hydroxyflavone, found in *M. calabura* extract, have a strong affinity for binding COX-2 as an inflammatory receptor in molecular dynamics studies, according to an in silico study by Tuwalaid et al. [22].

Inflammation is closely associated with COPD because the disease is related to inflammation of the respiratory system. Therefore, further investigation of anti-COPD in vitro is essential to produce a candidate herbal medicine supported by strong scientific evidence, along with the working mechanism, characteristic compounds, and active compounds. Based on the available literature, studies to develop H_2_O extracts derived from Wedelia (*Sphagneticola trilobata*) and Sembung Rambat (*Mikania micrantha*) have not been carried out. Ethanol extract of *M. micrantha* was examined by Deori et al. [25] in vivo, with the results showing significant inflammation-inhibiting properties. However, in vitro studies on *S. trilobata* and *M. micrantha* plants have not previously been conducted. Accordingly, the goal of this study is to employ in vitro methods to determine the key inflammatory mediator inhibitory activity, like IL-1β, IL-6, and IL-2, released from the aqueous extract of *S. trilobata* and *M. micrantha* on LPS-treated RAW 264.7 macrophages. The secondary metabolite profile was examined in each extract to determine which compounds act as inhibitors of inflammation related to the respiratory system. The results of this study can help the pharmaceutical industry achieve the mission to provide trusted health products to improve public health. It also supports the vision of becoming a leading health product manufacturer that dominates the market in Indonesia and Asia, with minimal side effects and by using natural raw materials from local sources.

## 2. Materials and Methods

### 2.1. Preparation of Raw Materials

Wedelia (*S. trilobata*) and Sembung Rambat (*M. micrantha*) were obtained from the Biopharmaceutical Cultivation Conservation Unit (UKBB) Garden at the Tropical Biopharmaceutical Study Center, IPB University. The plants were harvested at the age of 3–4 months by pruning the stems, and then the fresh leaves were made into simple drugs through a process of washing, drying in a 45 °C oven for 36 h, and grinding into powder using a grinder with an 80 mesh sieve. The extraction was carried out using the maceration method. The ratio of the plant powder to the solvent was 1:10, with distilled water used as the solvent at room temperature. The maceration process was carried out for 3 × 24 h. All macerate was collected and evaporated using a rotary evaporator to obtain a thick extract, which was then freeze-dried to obtain a dry extract.

### 2.2. Determination of Standardization of Raw Materials for Simplicia and Extracts

Post-harvest evaluation at the supplier/farmer level was conducted to evaluate the standardized post-harvest process for raw materials. The standardization was conducted in accordance with Indonesian Food and Drug Authority (BPOM/*Badan Pengawas Obat dan Makanan*) requirements. The analyses consist of moisture, ash, microbiological, and heavy metal content to prepare a Certificate of Analysis (CoA) for the raw materials.

Standardization of raw materials was carried out in accordance with BPOM requirements. Analysis carried out for the standardization of raw materials includes moisture content, ash content, acid-insoluble ash content, water-soluble extract content, ethanol-soluble extract content, microbiological content, and heavy metal content for lead (Pb) and cadmium (Cd). The procedures followed standard methods, including AOAC [26] and Indonesian National Standard (SNI) by BSN [27,28,29] as follows:

- *Moisture content*—A covered porcelain dish was heated in an oven at 105 °C for 30 min, cooled in a desiccator for 15 min, and weighed. Approximately 10 g of sample was accurately weighed into the pre-weighed dish and dried at 105 °C for 5 h. The dish containing the sample was then cooled in a desiccator for 30 min and weighed. Drying was continued at 1 h intervals until the difference between two consecutive weighings was not more than 0.25%. The moisture content was calculated using Equation (1).
  (1) $$Moisture\;content=\frac{W-(W_{1}-W_{2})}{W}\times 100\%$$
  $$\begin{array}{c} W=sample\;weight\;before\;drying\;\left(g\right)\\ W_{1}=sample+covered\;porcelain\;dish\;weight\;after\;drying\;\left(g\right)\\ W_{2}=heated\;empty\;covered\;porcelain\;dish\;weight\;(g) \end{array}$$
- *Ash content*—2–3 g of sample was accurately weighed into a porcelain crucible of known weight. The sample was charred over a burner flame, then ashed in an electric furnace at a maximum temperature of 550 °C until complete ashing (occasionally, the furnace door is opened slightly to allow oxygen to enter). The crucible and sample were cooled in a desiccator, then weighed until the weight remained constant. The ash content was calculated using Equation (2).
  (2) $$Ash\;content=\frac{W_{1}-W_{2}}{W}\times 100\%$$
  $$\begin{array}{c} W=sample\;weight\;before\;ashing\;\left(g\right)\\ W_{1}=sample+covered\;porcelain\;dish\;weight\;after\;ashing\;\left(g\right)\\ W_{2}=heated\;empty\;covered\;porcelain\;dish\;weight\;\left(g\right) \end{array}$$
- *Acid-insoluble ash content*—The ash obtained from the previous ash content determination was dissolved in 25 mL of 10% HCl and boiled for 5 min. The solution was then filtered through ashless filter paper and washed with distilled water until chloride-free. The filter paper was placed in a porcelain cup of known weight and dried in an oven, then ashed. The porcelain cup was cooled in a desiccator to room temperature, then weighed. The weighing was repeated until a constant weight was achieved. The acid-insoluble ash content was calculated using Equation (2).
- *Water- or ethanol-soluble extract content*—Five grams of sample were accurately weighed using a stoppered flask. The sample was macerated with 100 mL of water or ethanol (95%) while shaking for the first 6 h and then left for 18 h. The mixture was quickly filtered to avoid solvent evaporation. The filtrate was transferred to a 100 mL volumetric flask, calibrated, and shaken until homogeneous. Twenty milliliters of the solution were accurately taken and dried in an oven at 105 °C in a shallow dish of known weight. Next, it was cooled in a desiccator and weighed. The drying, cooling, and weighing procedures were repeated until a constant weight was obtained. The water or ethanol extract content was calculated using Equation (3).
  (3) $$Water\;or\;ethanol\;soluble\;extract\;content=\frac{W_{1}-W_{2}}{W}\times 100\%\times 5$$
  $$\begin{array}{c} W=sample\;weight\;before\;drying\;\left(g\right)\\ W_{1}=sample+dish\;weight\;after\;drying\;\left(g\right)\\ W_{2}=empty\;dish\;weight\;(g) \end{array}$$
- Microbial content—
  (i) *Total Plate Count (TPC)*: Initially, 1 g of the sample was weighed and thoroughly mixed into 10 mL of sterile water to create the stock suspension. A serial dilution was then performed to achieve the required concentration levels. From the prepared dilution tubes, aliquots of 1 mL and 0.1 mL were drawn and transferred into sterile Petri dishes. Subsequently, molten Plate Count Agar (PCA) or Nutrient Agar (NA) (Oxoid) was poured into the dishes to mix with the sample. Finally, the Petri dishes were incubated at 37 °C for 24 to 48 h to allow the microbial colonies to develop.
  (ii) *Coliform Test*: Initially, 1 g of the sample was weighed and introduced into 10 mL of sterile water. A serial dilution was then performed to obtain the required dilution series. From these dilutions, aliquots of 1 mL and 0.1 mL were drawn and transferred into sterile Petri dishes. Subsequently, Eosin Methylene Blue Agar (EMBA) (Oxoid) was poured into the dishes to mix with the sample. Finally, the Petri dishes were incubated at 25 °C for 24 h to allow for microbial observation.
  (iii) *Mold/Yeast Test*: Initially, 1 g of the sample was weighed and mixed into 10 mL of sterile water to create the stock suspension. A serial dilution was then performed to achieve the required dilution levels. From the prepared dilution tubes, aliquots of 1 mL and 0.1 mL were drawn and transferred into sterile Petri dishes. Subsequently, Potato Dextrose Agar (PDA) (Oxoid) was poured into the dishes to mix thoroughly with the sample. Finally, the Petri dishes were incubated at 30 °C for 2 to 4 days to allow for the growth and observation of mold and yeast colonies.
- *Heavy metal content for Pb and Cd*—Initially, 5 g of the sample was weighed and transferred into a porcelain dish or a 100 mL Pyrex beaker. The container was then moved into a muffle furnace at 200 °C, where the temperature was gradually increased to 500 °C over 2 h, and the sample was ashed overnight at 450–500 °C. Following this, the beaker was removed from the furnace and allowed to cool on an asbestos mat; if carbon residue remained, 1 mL of water and 2 mL of pro analyst HNO_3_ were added after cooling, dried over a water bath, and reheated at 500 °C for 1 h until a white ash was obtained. Next, 5 mL of HNO_3_ was added along the wall of the beaker, and the mixture was heated over a water bath until the ash was fully dissolved. The solution was quantitatively transferred into a 50 mL volumetric flask, diluted to volume with distilled water, and filtered using Whatman 540 filter paper. A blank was prepared concurrently using the same reagents. Subsequently, the absorption of the standard solutions, blank, and sample was measured using an atomic absorption spectrophotometer at specific wavelengths for each metal (217.0 nm for Pb and 228.8 nm for Cd). Finally, a calibration curve was constructed with absorbance plotted on the Y-axis and concentration (in $\frac{\mu g}{\mathrm{mL}}\;or\;\mathrm{ppm}$) on the X-axis, from which the metal content in the sample was calculated using Equation (4).
  (4) $$Heavy\;metal\;content\;(\mu g/g)=\frac{\mu g\;logam/mLfrom\;calibration\;curve\times V}{m}$$
  $$\begin{array}{c} V=dissolution\;volume\;\left(mL\right)\\ m=sample\;weight\;(g) \end{array}$$

### 2.3. LC-MS/MS Analysis

The H_2_O extracts’ chemical profiling from Wedelia and Sembung Rambat was conducted using LC-MS/MS on a UHPLC Vanquish system combined with a Thermo Scientific Q Exactive Plus Orbitrap HRMS. Potential constituents were assigned by searching the acquired spectral data against a laboratory-curated database using a maximum mass deviation of ±5 ppm. An Accucore C_18_ column (100 mm × 2.1 mm, 1.5 μm particle size) was employed for analytes’ chromatographic separation. The sample injection volume, flow rate, and column temperature were set at 5.0 μL, 0.2 mL/min, and 30 °C, respectively. The elution solvents comprised 0.1% formic acid in water (phase A) and 0.1% formic acid in acetonitrile (phase B). The gradient elution program consisted of 5%, 5–95%, 95%, and 5% solvent B over 0–1, 1–25, 25–28, and 28–33 min, respectively. The MS electrospray source was configured in negative-ion mode, with the spray voltage, capillary temperature, auxiliary and sheath gas flow rate set at 3.8 kV, 320 °C, 3 mL/min, and 15 mL/min, respectively. Fragmentation analysis employed collision energies of 18, 35, and 53 eV, and ions were acquired over an *m*/*z* range of 100–1500. Furthermore, LC-MS/MS data were analyzed through an in-house database to identify potential compounds and used a mass error of ±5 ppm. The identification of compounds was conducted by comparing fragmentation patterns and retention duration to data from an in-house database, and also various scientific journals [30].

### 2.4. RAW 264.7 Macrophage Cell Culture

Murine RAW 264.7 macrophage supplied by the Primate Research Center of IPB University (Bogor, Indonesia) served as the experimental cell line. The cells were regularly cultivated in DMEM (Dulbecco’s Modified Eagle’s Medium) supplemented with 1% penicillin-streptomycin and 10% FBS. Until sufficient confluence was reached, cell cultures were kept at 37 °C in a moist atmosphere with 5% CO_2_, after which cell detachment was performed using trypsin–EDTA (ethylenediaminetetraacetic acid) [20].

### 2.5. Cell Viability Assay

The MTT (3-(4,5-dimethylthiazol-2-yl)-2,5-diphenyltetrazolium bromide) technique was utilized for the cell viability test. For the assay, RAW 264.7 macrophages were plated in 96-well plates at 1 × 10^4^ cells/well initial density and they were then incubated for a full day. After replacing the rejected medium with a fresh one and adding it to the sample at 15.625, 31.25, and 62.50 ppm, the sample was incubated for a full day. After removing the medium, PBS was used to wash the cells. Each well received around 10 μL of a 5 mg/mL MTT solution, and then placed in an incubator for another four hours. One hundred microliters of DMSO were utilized to dissolve the formazan crystals. A microplate reader set to 570 nm was utilized to estimate uptake [20]. Cell viability percentage was defined utilizing Equation (5).

(5) $$\%\;Cell\;viability=\left[\frac{{Absorbance}_{sample}-{Absorbance}_{blank}}{{Absorbance}_{control}-{Absorbance}_{blank}}\right]\times 100\%$$

### 2.6. Pro-Inflammatory Cytokine Inhibitory Activity Assay

Using the ELISA technique, increased IL-6, IL-2, and IL-1β levels in LPS-treated RAW 264.7 macrophages were measured to assess cytokine-suppressive activity. Moreover, 96-well plates were filled with 1 × 103 RAW 264.7 macrophages per well, which were then cultured for a full day. After discarding the medium, a fresh one was applied to the specimen at the 31.25 ppm concentration. After two more hours of incubation, the specimens were activated with 1 μg/mL LPS followed by culturing for a full day. The medium was collected and agitated for 10 min at 2000 rpm.

The amounts of IL-6, IL-2, and IL-1β present in the cell culture supernatants were quantified utilizing Elabscience mouse ELISA kits by adhering to the supplier’s prescribed protocol [20]. The percentages of cytokines that promote inflammation inhibition were computed using Equation (6).

(6) $$\%\;inhibition=\left[\frac{{Concentration}_{negativecontrol}-{Concentration}_{sample}}{{Concentration}_{negativecontrol}}\right]\times 100\%$$

### 2.7. Statistical Analysis

One-way ANOVA and Tukey’s post hoc test at a 95% confidence level were used to analyze the data employing statistical methods, which were depicted as the three replicates ± the SD average. A *p*-value ≤ 0.05 indicates a significant difference between groups in the analyzed populations.

## 3. Results and Discussion

### 3.1. Extraction Results

Extraction is the technique of utilizing a solvent to separate secondary metabolites or bioactive components from plant materials. The yield is dependent on the pH, temperature, solvent polarity, sample composition, and extraction duration [30,31]. In this study, the H_2_O extract of Sembung Rambat had a higher yield (26.65%) than Wedelia (20.04%). Wedelia and Sembung Rambat were derived employing H_2_O solvent, as indicated in Table 1; hence, there is no discernible difference in the yields of the two extracts, and both belong to the same group, Asteraceae [32,33].

### 3.2. Standardization of Raw Materials for Simplicia and Extracts

To guarantee the medicinal plant extract’s quality and safety criteria, standardization is required. Standardization of raw materials is carried out in accordance with the Herbal Pharmacopoeia and BPOM Regulation Number 32 of 2019 concerning the Safety and Quality Requirements for Traditional Medicines. The results of standardization assays on the raw materials and extracts of Wedelia and Sembung Rambat (Tables S1 and S2) for moisture and ash content, as well as microbial contamination parameters, met the standards in accordance with the Herbal Pharmacopoeia and BPOM Regulation Number 32 of 2019 [34] concerning the Safety and Quality Requirements for Traditional Medicines. There is no standard reference pertaining to the acid-insoluble ash, water, and ethanol solubility of the Wedelia and Sembung Rambat plants and their extracts, but these data give information on the solvents used for further preparation of plant-based products.

Meanwhile, the analysis showed that the levels of Cd metal in the Wedelia and Sembung Rambat plants and water extracts exceeded the specified threshold. Naturally, Cd is found in sedimentary rocks, and the weathering of these rocks slowly releases Cd into the soil, which can be absorbed by plants. In addition, sources of heavy metal contamination can also come from added fertilizer base materials or from the environment. Heavy metals are often found in the use of organic and inorganic fertilizers and pesticides during cultivation [35]. Moreover, both plants have been reported to have the capacity as heavy metal bioremediators, which explains their correlation with high levels of cadmium [36,37]. The sample standardization analysis results of Wedelia and Sembung Rambat are illustrated in the Supplementary Materials (Tables S1 and S2). The data in this study indicate that selecting the right growing location and appropriate cultivation practices are important factors in reducing Cd content in plants. Furthermore, the extraction solvent’s choice is also important to minimize the transfer of Cd into the plant extract. These factors require greater attention in the development of herbal products to meet the safety standards. It is also recommended for future cultivation using soilless cultivation techniques to reduce heavy metal contamination in these two plants.

### 3.3. Putative Compounds in Wedelia and Sembung Rambat H_2_O Extracts

**Figure 1:**
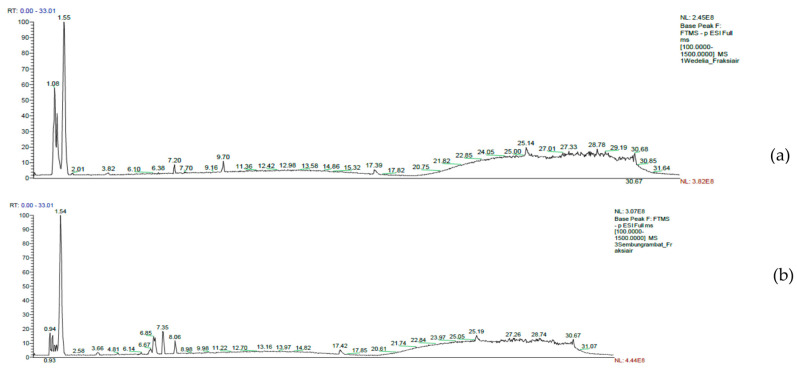
LC-MS/MS chromatogram for Wedelia H_2_O extract (**a**) and Sembung Rambat H_2_O extract (**b**).

The LC-MS/MS chromatogram for the H_2_O extracts of Wedelia and Sembung Rambat can be seen in Figure 1. This research found that amino acids, phenolic acids, and organic acids are 4 groups of compounds that are found in large quantities in both extracts. In addition, terpenoids were only found in the Wedelia H_2_O extract. The entire group of compounds (4 groups of compounds) consists of 17 compounds. It has been reported that all these groups of compounds function as anti-inflammatories, particularly by inhibiting the release of pro-inflammatory cytokines IL-6, TNF-α, and IL-1β (Table 2) [38,39,40,41]. Three compounds were also found together in both extracts, including L-(+)-Valine, 4-Aminobenzoic acid, and Protocatechuic acid.

### 3.4. Cell Viability

In order to analyze the anti-inflammatory properties of Wedelia and Sembung Rambat extracts, we initially measured their cytotoxicity in RAW 264.7 macrophages. This method is based on determining cell viability in cell culture to investigate the effects of pharmaceuticals in vitro on cell proliferation by cell-mediated cytotoxicity analysis. In this study, we used the MTT (3-(4,5-dimethylthiazol-2-yl)-2,5-diphenyltetrazolium bromide) assay. The principle of this method is to measure the mitochondrial dehydrogenase activity of living cells, which has the ability to convert MTT into formazan. The intensity of the purple color of formazan is determined using an absorbance value that is related to the number of living cells. The more intense the purple color, the higher the absorption value. This demonstrates that the higher the number of living cells reacting with tetrazolium salt, the more formazan is produced [19,50].

**Figure 2:**
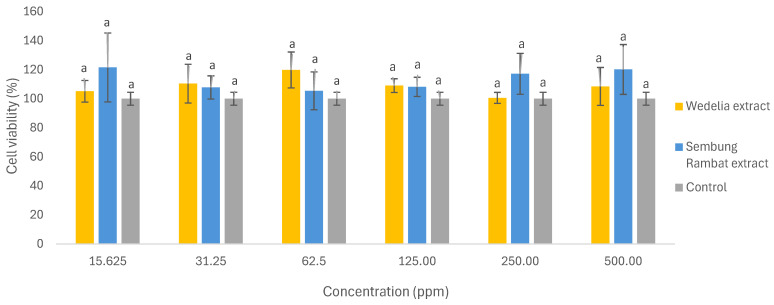
Viability of RAW 264.7 macrophage cells when treated with the extract. For each sample, values with different letters in the same column indicate significant differences at *p*-value ≤ 0.05 based on one-way ANOVA followed by Tukey’s test (*n* = 3). The results are sorted in ascending order: a > b > c > d > e.

The viability of RAW 264.7 macrophage cells when treated with H_2_O extracts of Wedelia and Sembung Rambat can be observed in Figure 2 and Table S3. This study used sample concentrations of 15.625, 31.25, 62.50, 125.00, 250.00, and 500.00 ppm. The cell viability assay is used to identify the concentration that does not cause macrophage cell death when treated with the optimum formula. A macrophage cell viability value of 264.7 is considered non-toxic if the cell viability reaches above 80% [51,52]. The results showed that the RAW 264.7 macrophage’s viability reached above 80% for all assay concentrations following treatment with aqueous extracts of Wedelia and Sembung Rambat. This result serves as the basis for determining safe concentration levels [53] in extract inhibition analysis against LPS-treated IL-6, IL-2, and IL-1β.

### 3.5. Inhibitory Activity of Single Extracts Against Pro-Inflammatory Cytokines

An extract concentration of 31.25 ppm was used in the inhibition assay of pro-inflammatory cytokine release from Wedelia and Sembung Rambat extracts. This concentration was selected because it is considered safe when converted to a human drug dose. Furthermore, the moderate concentration is expected to demonstrate effectiveness as a drug or inhibit the in vitro release of pro-inflammatory cytokines [54,55].

Tetrahydropyrimidine was used as the positive control because it is used as an anti-inflammatory drug (non-steroidal anti-inflammatory drugs/NSAIDs) that suppresses the production of pro-inflammatory cytokines and works by inhibiting inflammatory pathway enzymes such as COX-2 (cyclooxygenase-2) and iNOS (inducible nitric oxide synthase) [56,57].

The findings noted that in the IL-6 assay, the Sembung Rambat extract had the highest inhibition (84.03 ± 9.91%) and the lowest concentration (343.25 ± 9.91 pg/mL). In comparison, the Wedelia extract showed an IL-6 concentration of 544.222 ± 7.13 pg/mL with an inhibition of 74.68 ± 7.13%. Compared with both the negative and positive control groups, the two extracts demonstrated significantly different inhibitory effects. The positive ones explained an inhibition of 100.00 ± 0.00%, while the negative control demonstrated no inhibition of IL-6 cytokine release (0.00 ± 0.00%). The normal control did not show any IL-6 cytokine concentration or have an inhibition of 100.00 ± 0.00%.

In the IL-2 assay, the Sembung Rambat extract demonstrated the lowest concentration of 0.00 ± 0.00 pg/mL and the maximum inhibition of 100.00 ± 0.00%. This inhibition was similar to the reference treatment (100.00 ± 0.00%), with no significant difference. Meanwhile, the Wedelia extract had an IL-2 concentration of 7.88 ± 0.0 pg/mL and an inhibition of 67.15 ± 0.0. The negative control showed no inhibition of IL-2 cytokine release (0.00 ± 0.00%), and the normal control had a concentration of 6.55 ± 1.26 pg/mL or an inhibition of 72.72 ± 1.26%. However, the Sembung Rambat extract had greater inhibition of IL-2 cytokine release than the normal control.

**Figure 3:**
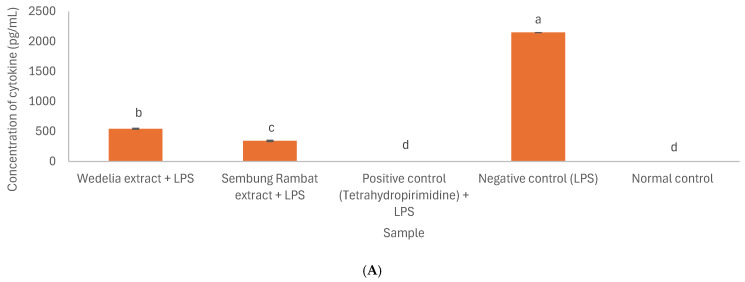

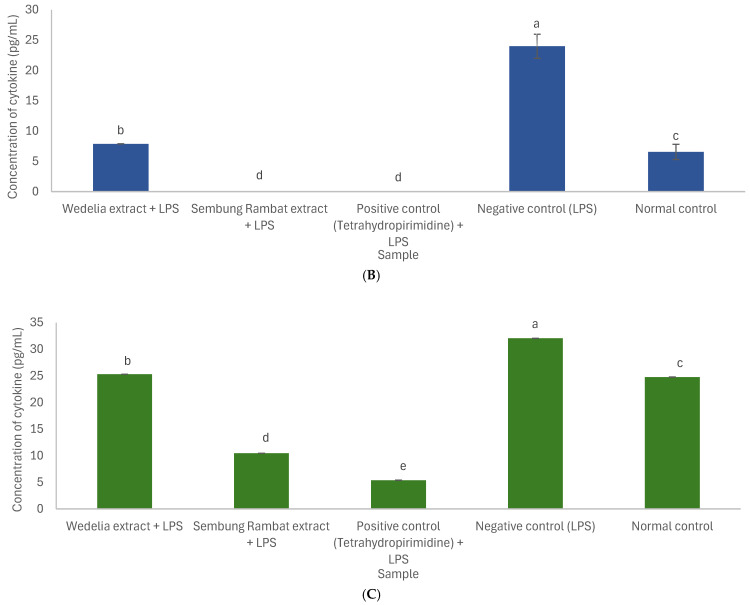
Concentrations of IL-6 (**A**), IL-2 (**B**), and IL-1β (**C**) produced by Wedelia and Sembung Rambat extracts. For each sample, values with different letters in the same column indicate significant differences at *p*-value ≤ 0.05 based on one-way ANOVA followed by Tukey’s test (n = 3). The results are sorted in ascending order: a > b > c > d > e.

**Figure 4:**
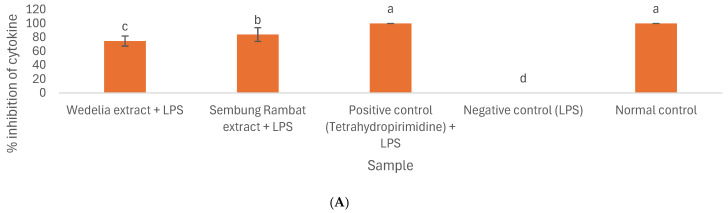

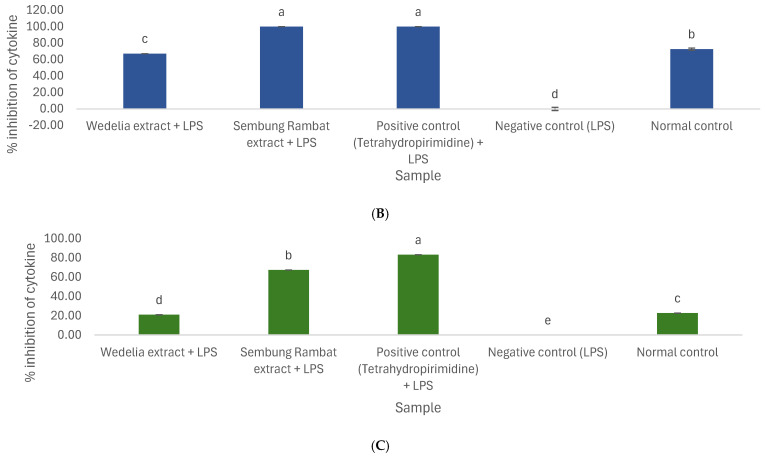
Inhibition of Wedelia and Sembung Rambat extracts against IL-6 (**A**), IL-2 (**B**), and IL-1ß (**C**). For each sample, values with different letters in the same column indicate significant differences at *p*-value ≤ 0.05 based on one-way ANOVA followed by Tukey’s test (n = 3). The results are sorted in ascending order: a > b > c > d > e.

In the IL-1β assay, the Sembung Rambat extract had the lowest concentration of 10.46 ± 0.00 pg/mL and the highest inhibition of 67.34 ± 0.00%. The Wedelia extract yielded an IL-1β concentration of 25.32 ± 0.00 pg/mL with an inhibition of 20.95 ± 0.00%. Meanwhile, the positive control had a higher inhibition than the Rambat extract, namely 83.16 ± 0.00%. The untreated control did not exhibit inhibition of IL-β cytokine release. The normal control showed an IL-1β concentration of 24.78 ± 0.00 pg/mL or 22.63 ± 0.00% inhibition. Nevertheless, the extract’s inhibition was still higher than the normal control. Based on the test, both extracts have the potential to inhibit pro-inflammatory cytokine production. Inhibition data from each extract, the positive control, the negative control, and the normal control are presented in Figure 3 and Figure 4, as well as Tables S4 and S5.

A major transcription factor in macrophages, the Nuclear Factor-kappa B (NF-κB) signaling pathway is necessary for the induction of many pro-inflammatory cytokine genes, including TNF-α, IL-1β, IL-6, IL-12, and COX-2. NF-κB activation occurs via two primary mechanisms: the classical (canonical) pathway and the alternative (non-canonical) pathway. The classical pathway is triggered by pro-inflammatory stimuli such as TNF-α, IL-1, and LPS. This pathway induces protein degradation, resulting in the release and translocation of NF-κB dimers to the cell nucleus, where they activate the expression of genes associated with inflammation and cellular defense. As this pathway operates rapidly and plays a dominant role in the acute inflammatory response, NF-κB is closely linked to the pathogenesis of inflammatory diseases such as COPD. Therefore, extracts of Wedelia and Sembung Rambat, which can inhibit the release of the pro-inflammatory cytokines IL-6, IL-2, and IL-1β, will have implications for the NF-κB signaling pathway [58,59].

The assay results are consistent with the metabolite profile data, showing that amino and phenolic acids dominate in reducing pro-inflammatory cytokine production. Previous studies have also confirmed these results. For example, Ellergezen et al. [43] reported that pregabalin, an amino acid, could inhibit the release of IL-6 and IL-2 in individuals with fibromyalgia syndrome (FMS) at a dose of 150 mg/day for at least three months. Furthermore, Li et al. [39] stated that Protocatechuic acid from the phenolic acids group at a dose of 0.25–1.0 µM can reduce the levels of IL-6, IL-1β, and TNF-α in LPS-induced macrophages.

Research related to the anti-inflammatory activity of Sembung Rambat extract has also been conducted by Deori et al. [25] in vivo, which stated that Sembung Rambat extract can reduce rat paw edema in carrageenan-induced acute and sub-acute inflammation at a dose of 200–400 mg/kgBW of Sembung Rambat ethanol extract. Furthermore, Samsuar et al. [60] also revealed that the ethanol fraction of Sembung Rambat at a dose of 450 mg/kgBW in vivo has anti-inflammatory activity on rat paw edema induced by carrageenan for acute inflammation.

An in vivo inflammation-inhibiting study related to inflammation in the respiratory tract was conducted by Castro et al. [61], who stated that IL-1β inhibition reduced rat lung inflammation induced by exposure to cigarette smoke. Furthermore, Widowati et al. [62] stated that *Camellia sinensis* L. extract reduced IL-6 expression in an ARDS experimental rat model. Lee et al. [63] also indicated that glycyrrhizin inhibited IL-6 lipopolysaccharide-induced chronic pulmonary damage in a rat model. Algheshairy et al. [64] also stated that *Citrus limon* juice can reduce IL-6 levels in a respiratory-irritated rat model. Several in vivo studies have shown that inhibition of pro-inflammatory cytokines can reduce inflammation in the respiratory tract, thus aligning with the in vitro studies we have currently conducted.

## 4. Conclusions

Despite the significant results, certain limitations must be acknowledged. Standardization of raw materials for simplicia and extracts was carried out in accordance with the requirements of BPOM Number 32 of 2019 concerning the Safety and Quality Requirements for Traditional Medicines. The Cd ion content is of particular concern because it exceeds the BPOM standard limit for simplicia and both extracts. This may be attributed to contamination originating from fertilizer inputs or environmental sources. Heavy metals are frequently detected in both organic and inorganic fertilizers as well as pesticides during cultivation [35]. The results indicate that determining the ideal growth site and using suitable cultivation techniques are crucial for lowering the amount of Cd in plants.

Metabolite profiling of the two extracts was conducted by LC-MS/MS using a Thermo Scientific Vanquish UHPLC coupled with a Q Exactive Plus Orbitrap high-resolution mass spectrometer. Although this instrument is putative, the compound determination has a mass error of ±5 ppm and compound fragmentation, which increases the reliability of the identified compounds. However, further studies should be conducted using standard compounds to validate the compounds obtained.

The standardization assay and in vitro studies of pro-inflammatory cytokine release inhibition (IL6, IL-2, and IL-1β) showed that Wedelia and Sembung Rambat extracts possess inflammation-inhibiting activity related to COPD. The Sembung Rambat water extract inhibited 100.00 ± 0.00% of IL-2 and 67.34 ± 0.00 of IL-1β cytokine release in LPS-treated macrophages. Meanwhile, the Wedelia water extract inhibited 84.03 ± 9.91% of IL-6 cytokine production. The extract concentration used in this in vitro assay for the inhibition of pro-inflammatory cytokine release was 31.25 ppm. This specific concentration was selected because the subsequent research aims to develop an herbal medicine. Therefore, a moderate concentration is required—one that can be converted into a therapeutic dosage and demonstrate efficacy as an anti-inflammatory inhibitor. Nevertheless, assays across the extract concentration range of 15.625–500 ppm are still required to provide a complete set of assay results.

The results are limited to in vitro conditions, and the observed inflammation-inhibiting effects cannot be directly extrapolated to therapeutic efficacy in patients with COPD respiratory tract inflammation. Therefore, further studies are needed to develop in vitro formulations of these two extracts, followed by in vivo evaluation using appropriate animal models and clinical investigations to determine the effectiveness in treating respiratory inflammation associated with COPD patients.

In conclusion, the extracts of Wedelia and Sembung Rambat fulfilled the standards for moisture and ash content, as well as microbial contamination parameters, which is in line with the standardization assays conducted in accordance with the Herbal Pharmacopoeia and BPOM Regulation Number 32 of 2019. The findings indicated that both aqueous extracts demonstrated substantial anti-inflammation activity through inhibition of pro-inflammatory cytokine release in LPS-treated RAW 264.7 macrophages. Amino compounds together with phenolic acids detected in the aqueous extract are considered potential contributors to its inflammation-inhibiting properties, particularly through suppression of pro-inflammatory cytokine release, including IL-6, IL-2, and IL-1β. Both the Wedelia and Sembung Rambat extracts have considerable potential for further development, either individually or in combination, as inflammation-inhibiting agents for COPD management, particularly through the reduction in inflammation in the respiratory system.

## Figures and Tables

**Table 1:** Yields of Wedelia and Sembung Rambat extracts.

Sample	Yield (%)
Wedelia	20.04
Sembung Rambat	26.65

**Table 2:** Data on compounds that have the potential to be inhibitors of pro-inflammatory cytokines in the H_2_O extracts of Wedelia and Sembung Rambat.

No.	Groups of Compounds	No.	Compounds	Wedelia H_2_O Extract	Sembung Rambat H_2_O Extract	Anti-Inflammatory Activity	Reference
1.	Amino acids	1.	L-(+)-Valine	✓	✓	The free amino acid composition contained in chicken liver hydrolysate-based supplements can reduce IL-6, TNF-α, and IL-1β inflammation in mice fed a long-term high-fat diet (HFD).	[38]
		2.	L-(+)-Leucine	-	✓	The free amino acid composition contained in chicken liver hydrolysate-based supplements can reduce IL-6, TNF-α, and IL-1β inflammation in mice fed a long-term high-fat diet (HFD).	[38]
		3.	DL-Phenylalanine	-	✓	The free amino acid composition contained in chicken liver hydrolysate-based supplements can reduce IL-6, TNF-α, and IL-1β inflammation in mice fed a long-term high-fat diet (HFD).	[38]
		4.	DL-Glutamic acid	-	✓	The free amino acid composition contained in chicken liver hydrolysate-based supplements can reduce IL-6, TNF-α, and IL-1β inflammation in mice fed a long-term high-fat diet (HFD).	[38]
		5.	4-Aminobenzoic acid	✓	✓	The free amino acid composition contained in chicken liver hydrolysate-based supplements can reduce IL-6, TNF-α, and IL-1β inflammation in mice fed a long-term high-fat diet (HFD).	[38]
		6.	DL-Tyrosine	-	✓	The free amino acid composition contained in chicken liver hydrolysate-based supplements can reduce IL-6, TNF-α, and IL-1β inflammation in mice fed a long-term high-fat diet (HFD).	[38]
		7.	L-Proline	✓	-	L-Proline-based cyclic dipeptides derived from Pseudomonas sp. decrease pro-inflammatory cytokines in vitro.	[42]
		8.	Pregabalin	-	✓	Pregabalin could inhibit the release of IL-6 and IL-2 in individuals with fibromyalgia syndrome (FMS) at a dose of 150 mg/day for at least three months.	[43]
2.	Phenolic acids	9.	Protocatechuic acid	✓	✓	Protocatechuic acid can reduce the levels of IL-6, IL-1β, and TNF-α in LPS-induced macrophages.	[39]
		10.	(E)-p-coumaric acid	-	✓	(E)-p-coumaric acid can lower the levels of IL-6, TNF-α, and IL-1β in liver tissue of bisphenol A-induced rats.	[44]
		11.	Chlorogenic acid	✓	-	Chlorogenic acid can reduce levels of IL-6, TNF-α, and IL-1β in severe acute pancreatitis rats.	[45]
		12.	Salicylic acid	-	✓	Salicylic acid can inhibit the production of IL-6 in LPS-stimulated keratinocytes.	[46]
3.	Organic acids	13.	Gentiopicrin	-	✓	Gentiopicrin reduces the levels of IL-6, TNF-α, and IL-1β in LPS-induced inflammation.	[40]
		14.	Sweroside	-	✓	Sweroside can reduce the production of IL-6, TNF-α, and IL-1β in vitro.	[47]
		15.	Citric acid	✓	-	Citric acid suppressed the secretion of IL-6, TNF-α, and IL-1β in an LPS-stimulated inflammation model of primary intestinal epithelial cells.	[41]
		16.	Arabic acid	✓	-	Arabic acid lowered the levels of IL-6, TNF-α, and IL-1β in mice treated with high-fat diet-induced obesity.	[48]
4.	Terpenoids	17.	Navenone A	✓	-	Navenone A can reduce the levels of IL-6, TNF-α, and IL-1β in vitro.	[49]

## Data Availability

The original contributions in this study are included in the article. Further inquiries can be directed to the corresponding author.

## Supplementary Materials

The following supporting information can be downloaded at: https://www.mdpi.com/article/10.3390/cimb48070720/s1.
