# Supplementary material for: Development of Wedelia (Sphagneticola trilobata) and Sembung Rambat (Mikania micrantha) Extracts as Herbal Medicine for Chronic Obstructive Pulmonary Disease (COPD)
Source: Curr Issues Mol Biol. 2026 Jul 15;48(7):720. doi: 10.3390/cimb48070720 (PMC13408239; doi:10.3390/cimb48070720)
Supplement: Supplementary file 1 [file cimb-48-00720-s001.zip › cimb-4380102-supplementary.pdf]

Supplementary material Table S1. Results of the standardization analysis of the wedelia and sembung rambat simplicia

Simplicia	Parameter	Result	Unit	Standard Reference#
Wedelia	Moisture content	8.29	%	≤10%
	Ash content	13.68	%	
	Acid-insoluble ash	3.48	%	
	Water soluble extract	12.94	%	
	Ethanol soluble extract	6.78	%	
	**Microbial contamination:**			
	Total Plate Count (TPC)^	2.5×10^4	CFU/g	$\leq 5 \times 10^7$ CFU/g
	Coliform	Negative	Nd	$\leq 10^2$ CFU/g
	Mold/yeast	1.0×10^4	CFU/g	$\leq 5 \times 10^5$ CFU/g
	**Heavy metal contamination:**			
	Pb	nd*	nd*	≤10 ppm
	Cd	1.68	ppm	≤0,3 ppm
Sembung rambat	Moisture	8.87	%	≤10%
	Ash	15.16	%	
	Acid-insoluble ash	1.32	%	
	Water soluble	11.20	%	
	Ethanol soluble	3.62	%	
	**Microbial contamination:**			
	Total Plate Count (TPC)^	1.6×10^4	CFU/g	$\leq 5 \times 10^7$ CFU/g
	Coliform	1.2×10^2	CFU/g	$\leq 10^2$ CFU/g
	Mold/yeast	9.5×10^2	CFU/g	$\leq 5 \times 10^5$ CFU/g
	**Heavy metal contamination:**			
	Pb	nd*	nd*	≤10 ppm
	Cd	2.04	ppm	≤0.3 ppm

#Standard Reference based on BPOM Regulation No. 32 of 2019 concerning Safety and Quality Requirements for Traditional Medicines
^TPC = Total Plate Count - laboratory method for counting the number of living microorganisms (bacteria) in a sampleU

Supplementary material Table S2. Results of the standardization analysis of the Wedelia and Sembung Rambat extracts

Extract	Parameter	Result	Unit	Standard Reference#
Wedelia (Water extract)	Moisture	9.34	%	≤10%
	Ash	18.04	%	
	Acid-insoluble ash	0.34	%	
	Water soluble	75.03	%	
	Ethanol soluble level	8.62	%	
	**Microbial contamination:**			
	Total Plate Count (TPC)^	1.3×10^3	CFU/g	$\leq 5 \times 10^7$ CFU/g
	Coliform	Negative	nd*	$\leq 10^2$ CFU/g
	Mold/yeast	1.9×10^2	CFU/g	$\leq 5 \times 10^5$ CFU/g
	**Heavy mctal Contamination:**			
	Pb	nd*	nd*	≤10 ppm
	Cd	1.76	ppm	≤0.3 ppm
Sembung rambat (Water extract)	Moisture	10.68	%	≤10%
	Ash	22.13	%	
	Acid-insoluble ash	0.36	%	
	Water soluble	71.01	%	
	Ethanol soluble level	7.86	%	
	**Microbial contamination:**			
	Total Plate Count (TPC)^	4.5×10^2	CFU/g	$\leq 5 \times 10^7$ CFU/g
	Coliform	Negative	nd*	$\leq 10^2$ CFU/g
	Mold/yeast	≤ 10	CFU/g	$\leq 5 \times 10^5$ CFU/g
	**Heavy metal contamination:**			
	Pb	nd*	nd*	≤10 ppm[2)]
	Cd	2.72	ppm	≤0.3 ppm[2)]

#Standard Reference based on BPOM Regulation No. 32 of 2019 concerning Safety and Quality Requirements for Traditional Medicines
^TPC = Total Plate Count - laboratory method for counting the number of living microorganisms (bacteria) in a sample
*not detected

Supplementary material Table S3. Data of viability of RAW 264.7 macrophage cells when given the extract.

Concentration of extract	% Viability cell		
	Wedelia extract	Sembung Rambat extract	Control
15.625	105.12±7.46[a]	121.55±23.70[a]	100.00±4.42[a]
31.25	110.43±13.32[a]	107.82±7.95[a]	100.00±4.42[a]
62.5	119.80±12.40[a]	105.45±13.04[a]	100.00±4.42[a]
125.00	109.02±4.72[a]	108.15±6.61[a]	100.00±4.42[a]
250.00	100.59±3.75[a]	117.16±14.09[a]	100.00±4.42[a]
500.00	108.47±13.09[a]	120.16±17.16[a]	100.00±4.42[a]

For each sample, values with different letters in the same column indicate significant differences at p-value ≤ 0.05 based on one-way ANOVA followed by Tukey's test (n = 3). Results are sorted in ascending order: a > b > c > d >e.

Supplementary material Table S4. Data of concentration of cytokine produced by Wedelia and Sembung Rambat extracts.

Extract	Concentration of cytokine (pg/mL)		
	IL-6	IL-2	IL-1β
Wedelia extract + LPS	544.22±7.13[b]	7.88±0.00[b]	25.32±0.00[b]
Sembung Rambat extract + LPS	343.25±9.91[c]	0.00±0.00[d]	10.46±0.00[d]
Positive control (Tetrahydropirimidine) + LPS	0.00±0.00[d]	0.00±0.00[d]	5.39±0.00[e]
Negative control (LPS)	2149.14±0.00[a]	23.99±1.98[a]	32.03±0.00[a]
Normal control	0.00±0.00[d]	6.55±1.26[c]	24.78±0.00[c]

For each sample, values with different letters in the same column indicate significant differences at p-value ≤ 0.05 based on one-way ANOVA followed by Tukey's test (n = 3). Results are sorted in ascending order: a > b > c > d>e.

Supplementary material Table S5. Data of inhibition of Wedelia and Sembung Rambat extracts against IL-6, IL-2, and IL-1β

Extract	% inhibition of cytokine		
	IL-6	IL-2	IL-1β
Wedelia extract + LPS	74.68±7.13[c]	67.15±0.00[c]	20.95±0.00[d]
Sembung Rambat extract + LPS	84.03±9.91[b]	100.00±0.00[a]	67.34±0.00[b]
Positive control (Tetrahydropirimidine) + LPS	100.00±0.00[a]	100.00±0.00[a]	83.16±0.00[a]
Negative control (LPS)	0.00±0.00[d]	0.00±1.98[d]	0.00±0.00[e]
Normal control	100.00±0.00[a]	72.72±1.26[b]	22.63±0.00[c]

For each sample, values with different letters in the same column indicate significant differences at p-value ≤ 0.05 based on one-way ANOVA followed by Tukey's test (n = 3). Results are sorted in ascending order: a > b > c > d>e.
